# Computerized Tomography Technique for the Investigation of the Maxillary First Molar Mesiobuccal Root

**DOI:** 10.1155/2013/614898

**Published:** 2013-09-08

**Authors:** Stefano Corbella, Massimo Del Fabbro, Igor Tsesis, Silvio Taschieri

**Affiliations:** ^1^Centre for Research in Oral Implantology, Department of Biomedical, Surgical and Dental Sciences, Università degli Studi di Milano, IRCCS Istituto Ortopedico Galeazzi, Milan, Italy; ^2^Centre for Research in Oral Health, Department of Biomedical, Surgical and Dental Sciences, Università degli Studi di Milano, IRCCS Istituto Ortopedico Galeazzi, Milan, Italy; ^3^Section of Endodontology, The Maurice and Gabriela Goldschleger School of Dental Medicine, Tel Aviv University, Israel

## Abstract

The aim of this paper was to review the literature about the use of computerized tomography to evaluate the presence and characteristics of the second mesiobuccal canal in the maxillary first molar. An electronic search was performed. Frequencies of the presence of second mesiobuccal canal and root anatomy characteristics were extracted from the selected studies. Pooled frequencies were calculated as weighted means. Seven articles were included. A second mesiobuccal canal was present in 59.32% of the teeth, and it was noncommunicating in 58.45% of teeth presenting the canal itself. The most common root canal morphology was single canal or two separated canals. The present paper showed that cone beam CT is a viable radiologic device for the evaluation of the mesiobuccal root of maxillary first molars. In fact, it was observed that the frequency of second mesiobuccal canal detection is similar to those presented by clinical studies or micro-CT evaluations.

## 1. Introduction

A sound knowledge of root canal anatomy is mandatory in order to perform an adequate root canal treatment. Studies reported that failure to detect all the canals present in a root canal system was one of the causes of failure of endodontic therapy [1–6].

A number of studies that evaluated the anatomy of mesiobuccal roots of maxillary permanent first molars reported a wide range of anatomical variations [7–10]. 

It was hypothesized that failure to detect, debride, and fill a second mesiobuccal canal (MB2) of first permanent maxillary molars was one of the main causes of poor long-term prognosis after root canal treatment in these teeth [11, 12]. 

While many *ex vivo* studies investigated the presence of a mesiobuccal canal using canal staining, cross-sectioning, and dentine examination through magnification devices [13–15], the most used technique to investigate the anatomy of these teeth prior to an endodontic treatment is periapical radiography, which does not allow a complete detailed evaluation of the root canal anatomy [16]. 

Cone-beam computed tomography (CBCT) was developed in the 1990s with the aim of producing maxillofacial three-dimensional images using a lower radiation dose than conventional computed tomography (CT) [17, 18]. The characteristics of the CBCT scanning were described as well suited to the endodontic field because of the higher accuracy of the device in comparison to that of the standard CT [17, 19]. 

Despite the known limitations of the CBCT (scattering [20], lower resolution than conventional radiography [17, 21]), it proved to be valuable in endodontic therapy for the diagnosis and evaluation of root canal anatomy [17]. 

The aim of the present paper was to systematically review the current scientific literature about the use of CT for the investigation of the mesiobuccal roots and canals of first permanent maxillary molars. 

## 2. Materials and Methods

### 2.1. Search Strategy

The following electronic databases were searched: MEDLINE (through PubMed interface http://www.ncbi.nlm.nih.gov/sites/pubmed), Scopus (http://www.scopus.com/), EMBASE (http://www.embase.com/), and the Cochrane Library (http://www.cochrane.org/). A search string was prepared ad hoc combining keywords with the use of Boolean operators “AND” and “OR”. The search string was (“Cone Beam Computed Tomography” OR “Cone Beam CT” OR “CBCT” OR “Computed Tomography” OR “CT”) AND (“endodontics” OR “endodontic diagnosis” OR “maxillary first molar” OR “fourth canal” OR “dental anatomy” OR “root canal” OR “mesiobuccal canal”). Results were limited by year of publication (from 1970 on), and the last search was performed in September 2012. In addition, a manual forward and backward search was performed in the reference lists of selected articles from the search results and of articles from the search results that were published in *Journal of Endodontics, International Endodontic Journal, Oral Surgery Oral Medicine Oral Pathology Oral Radiology and Endodontology, Journal of Dentistry, Journal of Dental Research, Clinical Oral Investigations, European Journal of Oral Sciences, Odontology, Dentomaxillofacial Radiology, Oral Radiology, Australian Dental Journal, *and* Australian Endodontic Journal.* No language restriction was posed.

### 2.2. Study Selection Criteria

The following inclusion criteria had to be met in order to be included in the review:

1. any study design (prospective or retrospective);
2. *ex vivo* or *in vivo* studies;
3. root canal anatomy evaluated using CT;
4. at least 10 teeth analyzed;
5. description of presence/absence of MB2 in maxillary first molars;
6. clear description of tooth type and location.

Studies not meeting the above criteria were excluded from the review.

### 2.3. Data Extraction and Analysis

The following parameters were recorded in an electronic form:

- presence/absence of the MB2 in maxillary first molars;
- any anatomical characteristics of the MB2, as described by Vertucci [29];
- methods for detection and CBCT machine characteristics.

Risk of bias was assessed for each of the included studies, considering the following parameters:

- *number of examined teeth*, posing that when less than 50 teeth were evaluated, the study had a moderate risk of bias; otherwise, the risk of bias for this parameter was judged as low;
- *quality of data reporting*, considering that if the authors did not report the characteristics of the root canal anatomy of MB2, but only the presence/absence of the canal, the study was judged at moderate risk of bias;
- *fulfillment of the aims of the study, reporting of individual data instead of frequency percentages, *and* clear description of population characteristics* were evaluated, and when missing, a moderate risk of bias was assigned.

Pooled data were analyzed through an evaluation of the weighted mean prevalence of MB2. Weighted means were calculated for the frequencies of different mesiobuccal canals morphologies following the Vertucci classification [29]. Moreover, a critical evaluation of differences among the various detection methods in terms of presence/absence of MB2 was performed. 

## 3. Results

**Figure 1:**
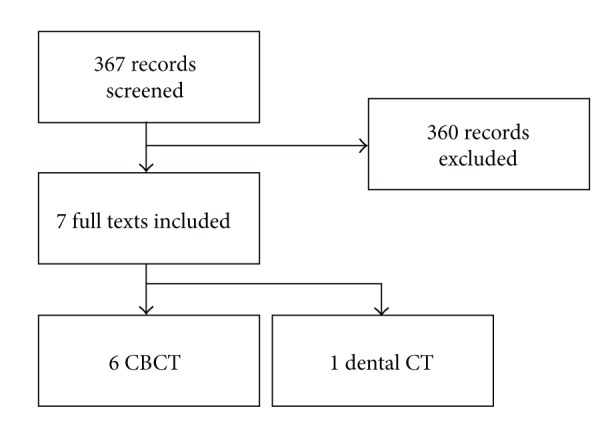
Article selection process.

Article selection process is summarized in the flowchart shown in Figure 1. The electronic search yielded 367 articles. After title and abstract screening, 20 articles were selected for full-text evaluation. The article selection was performed independently by two authors (SC and MDF). In case of disagreement, a joint decision was taken by discussion. A total of seven articles were finally included in the review: one article described the use of dental-CT *in vivo* [28], and six described the use of CBCT both in *in vivo* and in *ex vivo* studies [22–27]. Data about study design, population, and device used are summarized in Table 1. 

Each of the included studies was evaluated by two authors (SC and ST) for risk of bias analysis, whose results are presented in Table 2.

In pooled analysis, the second mesiobuccal canal was present in 59.32% of the examined teeth (out of a total of 1964 teeth), and it was evaluated as completely independent (noncommunicating) in 58.45% of the teeth presenting the canal (*N* = 1165). Data about frequency of the presence of the second mesiobuccal canal with different detection methods are summarized in Table 3.

Four studies presented data that could be classified according to the Vertucci classification of root canal morphology [24–27]. The data are summarized in Table 4. More than 37% of the classified 1741 teeth included in this analysis belonged to type IV, while 36.18% belonged to type I. These two types were the most frequently detected morphologies. 

Comparison of the different detection systems showed that in CBCT the MB2 was detected in 61.84% of teeth, while dental-CT showed MB2 presence in 39% of teeth.

## 4. Discussion

The present study was aimed to systematically review the root canal anatomy of the mesiobuccal roots of permanent maxillary first molars as shown in CT images.

The retrieved data showed that the detection of a separate mesiobuccal canal occurred in nearly two-thirds of the cases. The common occurrence of two canals in the mesiobuccal roots has a major implication in endodontics when performing a root canal therapy in maxillary first molars. 

A systematic review of the literature performed by Cleghorn and coworkers in 2006 [8]estimated the anatomy of the permanent maxillary first molar through a meta-analysis of data of about 8399 teeth from 34 laboratory studies and of 2576 teeth from 14 clinical studies. They reported that the incidence of two canals in the mesiobuccal root was 56.8%, which is similar to the results of the present study.

An issue to be considered is the age and gender of the subjects. Several authors found that a second mesiobuccal canal is less frequent in older subjects due to progressive calcification and obturation [8, 30–33]. As reported by other authors, there is conflicting evidence regarding differences related to gender of the subjects [11, 32]. 

The present study also showed that, if an MB2 was present it was mostly not merged with the primary one there were two separate apexes and two entrances without any merging (Vertucci Class IV [29]). This finding, which was also confirmed by previous systematic reviews [8, 9, 34], may have clinical relevance. The detection of a second mesiobuccal canal is mandatory for complete sterilization and filling of the root canal system, in order not to leave a pathway for bacterial migration towards the apex or a reservoir for microorganisms. 

Some studies investigated the use of magnification devices (microscope or magnifying loupes) as an adjuvant for the detection of a second mesiobuccal canal [34–37]. One of these studies showed that the MB2 was detected in 71.1% of maxillary first molars when using a microscope, in 62.5% of these teeth when using dental loupes, and only in 17.2% of these teeth without any magnification [36]. These results are similar to those reported in other studies and are comparable to the frequencies reported in the present review for CT.

However, the use of CBCT is considered very important for an adequate planning of endodontic surgery because of the capability to detect anatomical variations [17, 38]. The use of CBCT enables reproduction of three-dimensional anatomy, allowing the evaluation of bone thickness and the relationships between root apices, lesions, and anatomical structures such as the maxillary sinus [38–41]. 

In the present review, the included articles seemed to be very heterogeneous in population characteristics and study methodology, limiting the external validity of the results. Also, different CBCT devices were used, which should be considered as an important confounding factor, thus limiting the ability to perform a full meta-analysis.

Despite these limitations, the present study showed that CBCT may be useful in detecting the presence of a second mesiobuccal canal in permanent first molars. This can be considered as an important adjunct in pretreatment assessment for endodontic procedures, especially in periapical surgery.

## Figures and Tables

**Table 1:** Study characteristics.

Authors	Year	Study	Device	Characteristics (machine; voxel size)	Population	Patients/teeth
CBCT						
Filho et al. [22]	2009	*In vivo *	CBCT	i-CAT; 0.2 mm^3^	Brazil	NR/54
Blattner et al. [23]	2010	*In vitro *	CBCT	i-CAT; NR	—	NE/20
Neelakantan et al. [24]	2010	*Ex vivo *	CBCT	3D accuitomo; 0.125 mm^3^	India	NR/220
Zhang et al. [25]	2011	*In vivo *	CBCT	3D accuitomo; 0.125 mm^3^	China	269/299
Lee et al. [26]	2011	*In vivo *	CBCT	Volux; 0.167 mm^3^	Republic of Korea	276/458
Kim et al. [27]	2012	*In vivo *	CBCT	Dinnova; 0.167 mm^3^	Republic of Korea	415/814
Dental CT						
Rathi et al. [28]	2010	*In vivo *	Dental CT	Somatom; 1 mm^3^	India; age: 11–77 y	100/100

NR: Not reported; NE: Not estimable; MB: mesiobuccal.

**Table 2:** Risk of bias evaluation.

Authors	Year	Study	Number of teeth	Data reporting	Aims of the study	Individual data	Population characteristics
CBCT							
Filho et al. [22]	2009	*In vivo *	Low	Moderate	Low	Low	Low
Blattner et al. [23]	2010	*In vitro *	Moderate	Low	Low	Low	Low
Neelakantan et al. [24]	2010	*Ex vivo *	Low	Low	Low	Low	Low
Zhang et al. [25]	2011	*In vivo *	Low	Low	Low	Low	Low
Lee et al. [26]	2011	*In vivo *	Low	Low	Low	Low	Low
Kim et al. [27]	2012	*In vivo *	Low	Low	Low	Low	Low
Dental CT							
Rathi et al. [28]	2010	*In vivo *	Low	Low	Low	Low	Low

**Table 3:** Evaluation of the presence of second mesiobuccal canal.

Authors	*N*	Presence of MB			MB independent			MB1 merge MB2		
		*n*	%	Cumulative %	*n*	%	Cumulative %	*n*	%	Cumulative %	
CBCT											
Filho et al. [22]	54	21	38.89		1	4.76		20	95.24		
Blattner et al. [23]	19	11	57.89		NR	—		NR	—		
Neelakantan et al. [24]	220	99	45		85	85.86		12	12.12		
Zhang et al. [25]	299	156	52.17		109	69.87		22	14.1		
Lee et al. [26]	458	329	71.83		160	48.63		152	46.2		
Kim et al. [27]	814	510	62.65		326	63.92		165	32.35		

Total	1864	1126		**61.84**	681		**63.93**	371		**39.68**	

Dental CT											
Rathi et al. [28]	100	39	39		NR	—		NR	—		

Total	100	39		**39**	0		**0**	0		**0**	

	**1964**	**1165**		**59.32**	**681**		**58.45**	**371**		**31.85**	

*N*: total number of teeth; *n*: number of teeth belonging to a category; NR: not reported; Cumulative %: weighted mean proportion of teeth.

**Table 4:** Root canal morphology following the Vertucci classification [29].

CBCT											Authors	*N*	Vertucci classification								
T I	T II	T III	T IV	T V	T VI	T VII	T VIII	NC				
(1)	(2-1)	(1-2-1)	(2)	(1-2)	(2-1-2)	(1-2-1-2)	(3)					
Neelakantan et al. [24]	220	114	12	0	85	0	0	0	2	0
Zhang et al. [25]	299	113	22	0	109	25	0	0	0	0
Lee et al. [26]	458	129	152	0	160	11	0	0	6	0
Kim et al. [27]	814	292	164	2	326	16	1	0	0	0

Total		**648**	**350**	**2**	**680**	**52**	**1**	**0**	**8**	**0**

**%**		**36.18**	**19.54**	**0.11**	**37.97**	**2.90**	**0.06**	**0.00**	**0.45**	**0.00**

*N*: number of teeth.

## References

[1] Bernstein SD, Horowitz AJ, Man M (2012). Outcomes of endodontic therapy in general practice: a study by the practitioners engaged in applied research and learning network. *Journal of the American Dental Association*.

[2] Song M, Kim HC, Lee W, Kim E (2011). Analysis of the cause of failure in nonsurgical endodontic treatment by microscopic inspection during endodontic microsurgery. *Journal of Endodontics*.

[3] Faramarzi F, Fakri H, Javaheri HH (2010). Endodontic treatment of a mandibular first molar with three mesial canals and broken instrument removal. *Australian Endodontic Journal*.

[4] Ricucci D, Siqueira JF (2008). Anatomic and microbiologic challenges to achieving success with endodontic treatment: a case report. *Journal of Endodontics*.

[5] Weine FS, Healey HJ, Gerstein H, Evanson L (1969). Canal configuration in the mesiobuccal root of the maxillary first molar and its endodontic significance. *Oral Surgery, Oral Medicine, Oral Pathology*.

[6] Ricucci D, Siqueira JF (2010). Fate of the tissue in lateral canals and apical ramifications in response to pathologic conditions and treatment procedures. *Journal of Endodontics*.

[7] Ng YL, Aung TH, Alavi A, Gulabivala K (2001). Root and canal morphology of Burmese maxillary molars. *International Endodontic Journal*.

[8] Cleghorn BM, Christie WH, Dong CCS (2006). Root and root canal morphology of the human permanent maxillary first molar: a literature review. *Journal of Endodontics*.

[9] Kulid JC, Peters DD (1990). Incidence and configuration of canal systems in the mesiobuccal root of maxillary first and second molars. *Journal of Endodontics*.

[10] Stropko JJ (1999). Canal morphology of maxillary molars: clinical observations of canal configurations. *Journal of Endodontics*.

[11] Weine FS, Hayami S, Hata G, Toda T (1999). Canal configuration of the mesiobuccal root of the maxillary first molar of a Japanese sub-population. *International Endodontic Journal*.

[12] Wolcott J, Ishley D, Kennedy W, Johnson S, Minnich S, Meyers J (2005). A 5 yr clinical investigation of second mesiobuccal canals in endodontically treated and retreated maxillary molars. *Journal of Endodontics*.

[13] Alavi AM, Opasanon A, Ng YL, Gulabivala K (2002). Root and canal morphology of Thai maxillary molars. *International Endodontic Journal*.

[14] Weng XL, Yu SB, Zhao SL (2009). Root canal morphology of permanent maxillary teeth in the han nationality in Chinese Guanzhong area: a new modified root canal staining technique. *Journal of Endodontics*.

[15] Yoshioka T, Kikuchi I, Fukumoto Y, Kobayashi C, Suda H (2005). Detection of the second mesiobuccal canal in mesiobuccal roots of maxillary molar teeth ex vivo. *International Endodontic Journal*.

[16] Sherwood IA (2012). Pre-operative diagnostic radiograph interpretation by general dental practitioners for root canal treatment. *Dentomaxillofacial Radiology*.

[17] Patel S (2009). New dimensions in endodontic imaging—part 2: cone beam computed tomography. *International Endodontic Journal*.

[18] Schulze D, Heiland M, Thurmann H, Adam G (2004). Radiation exposure during midfacial imaging using 4- and 16-slice computed tomography, cone beam computed tomography systems and conventional radiography. *Dentomaxillofacial Radiology*.

[19] Scarfe WC, Levin MD, Gane D, Farman AG (2009). Use of cone beam computed tomography in endodontics. *International Journal of Dentistry*.

[20] Mora MA, Mol A, Tyndall DA, Rivera EM (2007). In vitro assessment of local computed tomography for the detection of longitudinal tooth fractures. *Oral Surgery, Oral Medicine, Oral Pathology, Oral Radiology and Endodontology*.

[21] Farman AG, Farman TT (2005). A comparison of 18 different x-ray detectors currently used in dentistry. *Oral Surgery, Oral Medicine, Oral Pathology, Oral Radiology and Endodontology*.

[22] Filho FB, Zaitter S, Haragushiku GA, de Campos EA, Abuabara A, Correr GM (2009). Analysis of the internal anatomy of maxillary first molars by using different methods. *Journal of Endodontics*.

[23] Blattner TC, George N, Lee CC, Kumar V, Yelton CDJ (2010). Efficacy of cone-beam computed tomography as a modality to accurately identify the presence of second mesiobuccal canals in maxillary first and second molars: a pilot study. *Journal of Endodontics*.

[24] Neelakantan P, Subbarao C, Ahuja R, Subbarao CV, Gutmann JL (2010). Cone-beam computed tomography study of root and canal morphology of maxillary first and second molars in an Indian population. *Journal of Endodontics*.

[25] Zhang R, Yang H, Yu X, Wang H, Hu T, Dummer PMH (2011). Use of CBCT to identify the morphology of maxillary permanent molar teeth in a Chinese subpopulation. *International Endodontic Journal*.

[26] Lee JH, Kim KD, Lee JK (2011). Mesiobuccal root canal anatomy of Korean maxillary first and second molars by cone-beam computed tomography. *Oral Surgery, Oral Medicine, Oral Pathology, Oral Radiology and Endodontology*.

[27] Kim Y, Lee SJ, Woo J (2012). Morphology of maxillary first and second molars analyzed by cone-beam computed tomography in a Korean population: variations in the number of roots and canals and the incidence of fusion. *Journal of Endodontics*.

[28] Rathi S, Patil J, Jaju PP (2010). Detection of mesiobuccal canal in maxillary molars and distolingual canal in mandibular molars by dental CT: a retrospective study of 100 cases. *International Journal of Dentistry*.

[29] Vertucci FJ (1984). Root canal anatomy of the human permanent teeth. *Oral Surgery Oral Medicine and Oral Pathology*.

[30] Burke FM, Samarawickrama DY (1995). Progressive changes in the pulpo-dentinal complex and their clinical consequences. *Gerodontology*.

[31] Morse DR (1991). Age-related changes of the dental pulp complex and their relationship to systemic aging. *Oral Surgery Oral Medicine and Oral Pathology*.

[32] Pattanshetti N, Gaidhane M, Al Kandari AM (2008). Root and canal morphology of the mesiobuccal and distal roots of permanent first molars in a Kuwait population—a clinical study. *International Endodontic Journal*.

[33] Al Shalabi RM, Omer OE, Glennon J, Jennings M, Claffey NM (2000). Root canal anatomy of maxillary first and second permanent molars. *International Endodontic Journal*.

[34] Neaverth EJ, Kotler LM, Kaltenbach RF (1987). Clinical investigation (in vivo) of endodontically treated maxillary first molars. *Journal of Endodontics*.

[35] Voorde HEV, Odendahl D, Davis J (1975). Molar 4th canals: frequent cause of endodontic failure?. *Illinois Dental Journal*.

[36] Buhrley LJ, Barrows MJ, BeGole EA, Wenckus CS (2002). Effect of magnification on locating the MB2 canal in maxillary molars. *Journal of Endodontics*.

[37] Nosonowitz DM, Brenner MR (1973). The major canals of the mesiobuccal root of the maxillary 1st and 2nd molars. *The New York Journal of Dentistry*.

[38] Rigolone M, Pasqualini D, Bianchi L, Berutti E, Bianchi SD (2003). Vestibular surgical access to the palatine root of the superior first molar: “low-dose cone-beam” CT analysis of the pathway and its anatomic variations. *Journal of Endodontics*.

[39] Patel S, Dawood A (2007). The use of cone beam computed tomography in the management of external cervical resorption lesions. *International Endodontic Journal*.

[40] Taschieri S, Fabbro MD, Corbella S, Weinstein T, Rosano G, Tsesis I (2011). Endoscopic minimally invasive management of a periradicular lesion invading the maxillary sinus. *Journal of Oral Science*.

[41] Oberli K, Bornstein MM, von Arx T (2007). Periapical surgery and the maxillary sinus: radiographic parameters for clinical outcome. *Oral Surgery, Oral Medicine, Oral Pathology, Oral Radiology and Endodontology*.

